# Prevalence of Carbapenem-Resistant Gram-Negative Infections in the United States Predominated by *Acinetobacter baumannii* and *Pseudomonas aeruginosa*

**DOI:** 10.1093/ofid/ofx176

**Published:** 2017-08-16

**Authors:** Bin Cai, Roger Echols, Glenn Magee, Juan Camilo Arjona Ferreira, Gareth Morgan, Mari Ariyasu, Takuko Sawada, Tsutae “Den” Nagata

**Affiliations:** 1 Shionogi Inc., Florham Park, New Jersey; 2 ID3C, Easton, Connecticut; 3 Premier Research Service, Inc., Charlotte, North Carolina; 4 Shionogi & Co., Ltd., Kitaku, Osaka, Japan

**Keywords:** carbapenem resistance, Gram-negative, nonfermenters, CRE, length of stay, in-hospital mortality

## Abstract

**Background:**

Carbapenem-resistant (CR) Gram-negative pathogens are recognized as a major health concern. This study examined the prevalence of infections due to 4 CR Gram-negative species (*Acinetobacter baumannii, Pseudomonas aeruginosa*, *Klebsiella pneumoniae,* and *Escherichia coli*) in the United States and assessed their impact on hospital stays and mortality.

**Methods:**

Hospitalized patients with laboratory-confirmed infection due to any of the 4 Gram-negative pathogens were identified from the Premier Healthcare Database. Proportions of CR were calculated by pathogen and infection site (blood, respiratory, urinary, or other) for the United States as whole and by census regions. Crude and adjusted odds ratios for in-hospital mortality were produced using logistic regression.

**Results:**

From 2009 to 2013, 13 262 (4.5%) of 292 742 infections due to these 4 Gram-negative pathogens were CR. Of these CR infections, 82.3% were caused by *A. baumannii* (22%) or *P. aeruginosa* (60.3%), while 17.7% were caused by *K. pneumoniae* or *E. coli*. CR patients had longer hospital stays than carbapenem-susceptible (CS) patients in all pathogen-infection site cohorts, except in the *A. baumannii*-respiratory cohort. The crude all cause in-hospital mortality was greater for most pathogen-infection site cohorts of the CR group compared with the CS group, especially for *A. baumannii* infection in the blood (crude odds ratio [OR], 3.91; 95% confidence interval [CI], 2.69–5.70). This difference for the *A. baumannii*-blood cohort remained after adjusting for the relevant covariates (adjusted OR, 2.46; 95% CI, 1.43–4.22).

**Conclusion:**

The majority of CR infections and disease burden in the United States was caused by nonfermenters *A. baumannii* and *P. aeruginosa*. Patients with CR infections had longer hospital stays and higher crude in-hospital mortality.

Infections due to carbapenem-resistant (CR) Gram-negative pathogens have been reported from many countries with variable prevalence and associated morbidity and mortality [1–4]. The dramatic increase and spread of CR infections over the past decade are recognized as a major public health concern [2, 5–9].

Recent infection control measures have focused on the identification and spread of CR Enterobacteriaceae (CREs), which is mainly due to mobile carbapenemase enzymes, both serine (KPC, GES, and OXA) and metallo-carbapenemases (NDM, VIM, and IMP) [6, 10]. Resistance mechanisms involving porin channel mutations and efflux pump overproduction may also contribute to the identification of CREs [6, 10, 11]. The frequency of carbapenem resistance among the non-glucose fermenting *Pseudomonas aeruginosa* and *Acinetobacter baumannii* has also increased in recent years, but it was not well appreciated as a source of resistance transmission until 2017 [7, 12–16], when the World Health Organization recognized the importance of CR *A. baumannii* and *P. aeruginosa* as equal to that of the CREs [17]. These CR nonfermenters are often multidrug resistant and associated with substantial morbidity and mortality [18, 19]. This study was designed to determine the prevalence of CR organisms (CROs) in the United States, specifically the Enterobacteriaceae *Escherichia coli* and *Klebsiella pneumoniae* and the nonfermenters *Pseudomonas aeruginosa* and *Acinetobacter baumannii,* using an electronic database from a geographically diverse network of US hospitals. Besides determining the prevalence of CR infections in the United States, the study compared similar infections that were carbapenem susceptible (CS) in order to assess the mortality attributed to the CROs.

## METHODS

This retrospective study of CR Gram-negative infections between 2009 and 2013 was conducted using microbiology data linked with patient-level in-hospital discharge data from the Premier Healthcare Database [20]. The Premier Healthcare Database is an anonymous census of inpatients and hospital-clinic outpatients from geographically diverse, mixed teaching and nonteaching hospitals with varied bed sizes in the United States. The hospital discharge data contain information on admission, discharge, and a date-stamped log of all billed items, including procedures, medications, laboratory, diagnostic, and therapeutic services at the individual patient level.

Microbiology data, collected from approximately 30% of participating hospitals, include the time the microbiological culture was obtained, type of pathogen identified, and antimicrobial drugs used for susceptibility testing along with the method, result, and interpretation of antibiotic susceptibility. All data were determined by individual hospital laboratories, which were Clinical Laboratory Improvement Amendments (CLIA) compliant using standard laboratory methods, and interpretations of susceptibility results were reported as resistant (R), intermediate (I), susceptible (S), or none (N).

The pathogens were further identified by infection sites (blood, respiratory, urinary, or other) based on the source of the culture sample. Thus, this study had 16 distinct pathogen-infection site cohorts. Within each cohort, pathogens were characterized as CR or CS. Bacteria were defined as CR if they were resistant to at least 1 of the carbapenems tested and as CS if the pathogen was susceptible to or intermediate for the carbapenems tested. If selected patients had multiple hospitalizations during the study period, only data from the first hospitalization were used. If a microbiologic culture identified multiple pathogens, the patient was counted in each pathogen group. If a patient had multiple cultures of the same pathogen, the first culture was used as index date if the pathogen had the same susceptibility test results for all cultures. Otherwise, the first CR culture was used as the index date, and the patient was in the CR group. Only cultured pathogens from patients who were receiving antibiotic treatment were included in the final data set.

The primary outcome variables were the rates of CR in each pathogen-infection site cohort for all hospitals and separately for each US census region. Other outcome variables included in-hospital mortality, total length of stay (LOS) in the hospital, LOS after positive culture (iLOS), total LOS in the intensive care unit (ICU) for the CR group and CS group, and survival status within each pathogen-infection site cohort.

The use of antibiotics with Gram-negative activity was assessed by class (aminoglycoside, carbapenem, cephalosporin, colistin, monobactam, penicillin, polymyxin B, quinolone, tetracycline, and other Gram-negative antibiotics) and the time when the patient received them during the hospitalization. Antibiotics administered in the days prior to the date of the index culture were considered “prior” antibiotics, those received from the date of the index culture through 3 days after the index culture were considered “empiric,” and those received after 3 days from the index culture were considered “definitive” treatment.

### Study Population

All hospitalized patients with microbiological cultures identifying *A. baumannii, P. aeruginosa, K. pneumoniae,* or *E. coli* from appropriate clinical specimens and with associated interpretations of susceptibility to carbapenem antibiotics (doripenem, ertapenem, imipenem, meropenem) from patients who received at least 1 systemic antibiotic treatment were selected.

### Statistical Analyses

Categorical variables were described with the number of patients and percentages and compared between CR and CS in each pathogen-infection site cohort using a chi-square or Fisher’s exact test where appropriate. Continuous variables were described with mean, standard deviation, and median. The differences between CR and CS groups were analyzed using Student’s *t* test or the Wilcoxon rank-sum test. A *P*-value <.05 indicated statistically significant differences between groups.

In-hospital mortality was compared between CR and CS for each pathogen infection site cohort with and without adjusting for covariates, such as age, gender, race, ethnicity, mechanical ventilation, renal impairment, geographic region, and each of the Charlson comorbid conditions [21].

All analyses were performed using SAS 9.3 (SAS Institute Inc., Cary, North Carolina).

## RESULTS

This study includes data collected from 206 acute care hospitals in the United States between January 1, 2009, and December 31, 2013. The total numbers of pathogen isolates were 173 200 *E. coli,* 56 552 *K. pneumoniae*, 56 477 *P. aeruginosa*, and 6508 *A. baumannii*.

### Carbapenem Resistance Rate

**Figure 1.**
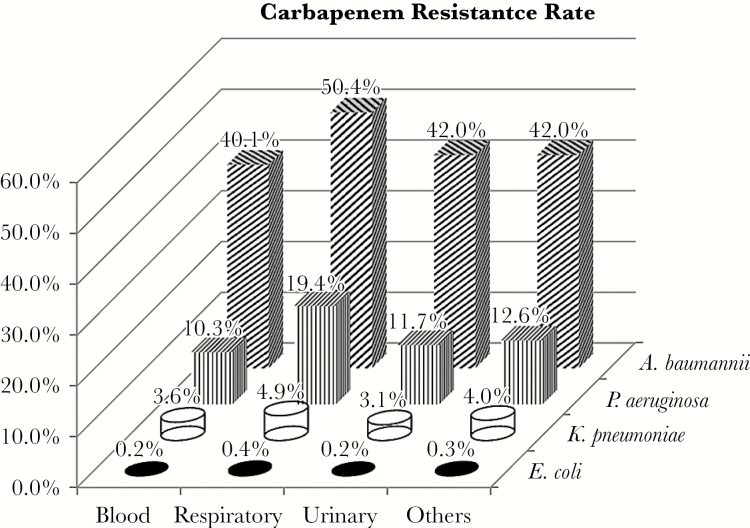
Carbapenem resistance rate by pathogen-infection site cohort.

The overall CR rate among the 4 selected pathogens was 4.5% (13 262 of 292 742). *A. baumannii* had the highest CR rate: blood 40.1%, respiratory 50.4%, urine 42.0%, and other 42%. The CR rate for *P. aeruginosa* ranked second highest and was more variable by infection site: 10.3% in blood, 19.4% in respiratory, 11.7% in urinary, and 12.6% in other (Figure 1). More than 80% of all CR infections were caused by *A. baumannii* or *P. aeruginosa*. This was consistent across 9 US census regions, except for the Middle Atlantic region, where 67% (Table 1) of CR infections were caused by the nonfermenters. For most regions, CR *P. aeruginosa* outnumbered CR *A. baumannii* by 3:1, except for the New England region, where 85% of CR infections were due to *P. aeruginosa* and only 6.4% were from *A. baumannii* (Table 2).

**Table 1.** Carbapenem-resistant infections caused by *A. baumannii, P. aeruginosa, K. pneumoniae*, and *E. coli* by US census region

US Census Regions	Number of Hospitals	All 4 Pathogens			*A. baumannii + P. aeruginosa*				*K. pneumoniae + E. coli*			
		Total Infections	CR Cases		Total Infections	CR Cases			Total Infections	CR Cases		
			Total CR Cases	% of Total Infections		CR Cases	% of Total Infections	% of Total CR Cases for 4 Pathogens		CR Cases	% of Total Infections	% of Total CR Cases for 4 Pathogens
East North Central	43	47 702	3164	6.6	10 381	2542	24.5	80.3	37 321	622	1.7	19.7
East South Central	9	21 440	770	3.6	4293	703	16.4	91.3	17 147	67	0.4	8.7
Middle Atlantic	19	37 582	2499	6.6	10 449	1672	16.0	66.9	27 133	827	3.0	33.1
Mountain	15	8582	765	8.9	2260	710	31.4	92.8	6322	55	0.9	7.2
New England	9	11 964	346	2.9	2417	316	13.1	91.3	9547	30	0.3	8.7
Pacific	27	39 045	1237	3.2	6447	1070	16.6	86.5	32 598	167	0.5	13.5
South Atlantic	40	62 217	2385	3.8	14 652	2017	13.8	84.6	47 565	368	0.8	15.4
West North Central	18	17 854	403	2.3	3130	329	10.5	81.6	14 724	74	0.5	18.4
West South Central	26	46 356	1693	3.7	8955	1550	17.3	91.6	37 401	143	0.4	8.4
Total	206	292 742	13 262	4.5	62984	10 909	17.3	82.3	229 758	2353	1.0	17.7

Abbreviations: CR, carbapenem resistant.

**Table 2.** *A. baumannii* and *P. aeruginosa* CR Infection by Region

Regions	Number of Hospitals	All 4 Pathogens			*Acinetobacter baumannii*				*Pseudomonas aeruginosa*			
		Total Infections	CR Cases		Total Infections	CR Cases			Total Infections	CR Cases		
			Cases	% of Total Infections^a^		Cases	% of Total Infections	% of Total CR Cases for 4 Pathogens		Cases	% of Total Infections	% of Total CR Cases for 4 Pathogens
East North Central	43	47 702	3164	6.6	1360	863	63.5	27.3	9021	1679	18.6	53.1
East South Central	9	21 440	770	3.6	498	223	44.8	29.0	3795	480	12.6	62.3
Middle Atlantic	19	37 582	2499	6.6	914	288	31.5	11.5	9535	1384	14.5	55.4
Mountain	15	8582	765	8.9	326	203	62.3	26.5	1934	507	26.2	66.3
New England	9	11 964	346	2.9	183	22	12.0	6.4	2234	294	13.2	85.0
Pacific	27	39 045	1237	3.2	739	327	44.2	26.4	5708	743	13.0	60.1
South Atlantic	40	62 217	2385	3.8	1322	488	36.9	20.5	13 330	1529	11.5	64.1
West North Central	18	17 854	403	2.3	256	77	30.1	19.1	2874	252	8.8	62.5
West South Central	26	46 356	1693	3.7	909	424	46.6	25.0	8046	1126	14.0	66.5
Total	206	292 742	13262	4.5	6507	2915	44.8	22.0	56 477	7994	14.2	60.3

Abbreviation: CR, carbapenem resistant.

### Demographic and Clinical Characteristics

Compared with patients with CS *A. baumannii*, patients with CR isolates tended to be older when the source was blood stream infection (mean±SD, 60.5 ± 16.6 years vs 53.6 ± 21.8 years), respiratory infection (mean±SD, 62.5 ± 16.8 vs 55.8 ± 23.5 years), or other infection sites (mean±SD, 60.7 ± 16.5 vs 57.0 ± 20.0 years), but of similar age when the source was urinary tract infection (mean±SD, 61.8 ± 17.5 vs 61.5 ± 19.8 years). More than two-thirds of patients with *A. baumannii* infections were white; however, the proportions of nonwhite patients in the CR groups were higher than in the CS groups for all infection sites. Slightly more male patients than female patients were identified in both the CR and CS groups. Nearly half of all patients with *A. baumannii* infections came to the hospitals from their homes, though the proportion was slightly higher in the CS group than in the CR group. Nearly 70% of these patients were admitted via emergency rooms. Patients with CR infections were more likely to be discharged to skilled nursing facilities or rehabilitation or long-term care facilities than patients with CS infections (43% vs 24% for blood, 51% vs 42% for respiratory, 58% vs 33% for urinary, and 51% vs 31% for other). Patients with CR infections also had a higher Charlson comorbidity index score for all infection sites, a longer total LOS for infections in the blood, urinary, and other (median days of CR vs CS, 15 vs 9 for blood, 12 vs 7 for urinary, and 12 vs 8 for other), and longer total ICU stays for blood, urinary, and other (median days of CR vs CS, 8 vs 5, 8 vs 4, and 9 vs 8, respectively). Similarly, a longer LOS after positive culture was seen in the CR group than the CS group among all patients and those who survived for each infection site except respiratory (Table 3). Among nonsurvivors with blood stream infection, the median LOS after positive culture was 2 days after the culture was obtained in the CR group and 4 days in the CS group. Patients with CR infections were slightly more likely to be on dialysis or have renal impairment during their hospital stays.

**Table 3.** Median Days Stayed in the Hospital After Positive Culture by Pathogen, Infection Site, and Survival Status

Pathogen	Length of Stay After Positive Culture	Blood		Respiratory		Urinary		Other	
		CR	CS	CR	CS	CR	CS	CR	CS
*Acinetobacter baumannii*	iLOS	8	6	8	10	8	4	7	5
	iLOS among survived	11	6	9	10	8	4	7	5
	iLOS among deceased	2	4	6	7	6	6	7	7
*Pseudomonas aeruginosa*	iLOS	5	4	20	7	5	3	8	5
	iLOS among survived	6	4	20	8	5	3	6	5
	iLOS among deceased	3	2	18.5	4	7	4	33.5	6
*Klebsiella pneumoniae*	iLOS	10	6	10	7	6	4	9	6
	iLOS among survived	10	6	11	8	6	4	9	6
	iLOS among deceased	6	3	6	4	8	4	9	6
*Escherichia coli*	iLOS	10	6	10	6	7	4	10	5
	iLOS among survived	10	7	10	6	6	4	9	5
	iLOS among deceased	9	1	11	5	9	5	14	6

Abbreviations: CR, carbapenem resistant; CS, carbapenem susceptible; iLOS, length of hospital stay after positive culture.

Similar patterns in admission sources, discharge status, hemodialysis, and renal impairment were observed for patients infected with CR *P. aeruginosa,* CR *K. pneumoniae*, and CR *E. coli* compared with their CS counterparts. However, patients with CR *P. aeruginosa* isolates were younger than patients with CS isolates for all infection sites (mean ± SD, 60.6 ± 17.3 vs 65.2 ± 19.1 for blood, 60.6 ± 19.1 vs 63.2 ± 20.3 for respiratory, 67.1 ± 17.1 vs 71.5 ± 17.1 for urinary, and 60.6 ± 18.5 vs 61.1 ± 20.2 for other), but they had similar Charlson comorbidity index scores. LOS, ICU LOS, and LOS after positive culture were longer for CR infections than CS infections for all infection sites.

The average age of patients with *K. pneumoniae* was approximately 60 years. There was no significant age difference between patients with CR and CS infections in blood, urine, or respiratory. In other infection sites, CR pathogen–infected patients were approximately 3 years older than those in the CS group.

There were more male patients than female patients in all study cohorts, except for those in the urinary cohort, where significantly higher numbers of female patients were identified. Due to the small number of patients in the CR-urinary cohort, there was no statistical significance in the observed differences in age (average of 60 years), race, or Charlson comorbidity index score.

### In-Hospital Mortality

Patients with CR infections consistently had numerically higher crude mortality rates than patients with CS infections for each of the 16 pathogen-infection site cohorts. The crude odds ratio (OR) for CR vs CS ranged from 1.31 for *E. coli* in respiratory to 3.91 for *A. baumannii* in blood (Table 4). After adjusting for age, gender, race, ethnicity, Charlson comorbid conditions, mechanical ventilation, renal impairment, and geographic census regions, the odds of dying from CR *A. baumannii* in blood (adjusted OR, 2.46) and in respiratory (adjusted OR, 1.27) and from *P. aeruginosa* in other (adjusted OR, 1.20) remained statistically significant (Table 4).

**Table 4.** In-Hospital Mortality: Crude and Adjusted Odds Ratio (CR vs CS)

Pathogen	Site	In-Hospital Mortality by Each Site, No. (%)		Crude			Adjusted^a^		
		CR	CS	Odds Ratio	Lower 95% CI	Upper 95% CI	Odds Ratio	Lower 95% CI	Upper 95% CI
*Acinetobacter baumannii*	Blood	103 (38.2)	55 (13.6)	3.91	2.69	5.70	2.46	1.43	4.22
	Respiratory	305 (26.0)	230 (19.9)	1.41	1.16	1.71	1.27	1.01	1.58
	Urinary	35 (9.6)	31 (6.2)	1.62	0.98	2.69	0.62	0.32	1.19
	Other	173 (15.6)	117 (7.7)	2.24	1.74	2.87	1.19	0.88	1.61
*Pseudomonas aeruginosa*	Blood	112 (33.3)	590 (20.1)	2.02	1.58	2.58	1.19	0.88	1.63
	Respiratory	661 (20.6)	1977 (14.8)	1.50	1.36	1.65	1.11	1.00	1.24
	Urinary	208 (9.8)	1031 (6.3)	1.58	1.35	1.85	1.05	0.88	1.26
	Other	389 (16.8)	1139 (7.1)	2.64	2.33	2.99	1.20	1.03	1.39
*Klebsiella pneumoniae*	Blood	63 (27.3)	819 (13.2)	2.46	1.83	3.32	1.10	0.75	1.61
	Respiratory	98 (25.4)	1547 (20.6)	1.32	1.04	1.67	0.97	0.76	1.25
	Urinary	90 (9.1)	1616 (5.2)	1.81	1.45	2.27	1.07	0.83	1.37
	Other	76 (18.1)	863 (8.7)	2.33	1.80	3.01	1.02	0.76	1.37
*Escherichia coli*	Blood	5 (16.1)	1709 (8.7)	2.03	0.78	5.29	1.91	0.60	6.10
	Respiratory	8 (26.7)	1469 (21.7)	1.31	0.58	2.95	0.74	0.29	1.85
	Urinary	13 (6.9)	4699 (3.9)	1.84	1.05	3.23	1.55	0.80	3.00
	Other	8 (10.1)	1466 (5.8)	1.84	0.89	3.84	0.78	0.33	1.84

Abbreviations: CI, confidence interval; CR, carbapenem resistant; CS, carbapenem susceptible.

^**a**^Adjusted for age, gender, race, ethnicity, various comorbid conditions, mechanical ventilation, renal impairment, and geographic regions.

Patients with CR pathogens were more likely to receive more than 1 systemic antibiotic than patients with CS pathogens. Also, patients with pathogens identified from the blood, respiratory, and other were more likely to receive 2 or more antibiotics than if the source was urinary. The number and types of antibiotics used were highly variable. During the definitive treatment period, more than 100 combinations (defined as different antibiotic classes regardless of the initiation time, dose, or duration) were used for each pathogen, but fewer than 20 of these combinations were used by more than 3% of patients. Fewer different antibiotic combinations were used to treat patients with CS infections than patients with CR infections.

## DISCUSSION

This study showed that in the United States the overall disease burden and impact on survival was greatest among the non-glucose fermenting *A. baumannii* and *P. aeruginosa*. *A. baumannii* had the highest rate of CR at each infection site, followed by *P. aeruginosa* and *K. pneumoniae*. *E. coli* consistently had the lowest rate of CR. The combined number of CR *A. baumannii* and CR *P. aeruginosa* infections accounted for more than 80% of all CR infections. Though this study included both community-onset and hospital-acquired infections, the pattern of CR from the selected 4 pathogens was comparable with the US Centers for Disease Control and Prevention’s (CDC’s) National Healthcare Safety Network (NHSN) results, which focused on health care–acquired infections via central lines, urinary catheters, and mechanical ventilators [9, 22]. The relative importance of CR *A. baumannii* has also been recently emphasized by the European Centre for Disease Prevention and Control (ECDC), which reported that CR *A. baumannii* (resistant through the production of carbapenemases) might be more widely disseminated in Europe than CREs [7, 12].

This study also showed that distribution of CR pathogens differed by infection sites. Respiratory was the most common site of CR infection for *A. baumannii* (40.3%) and *P. aeruginosa* (40.1%), while urinary was the most common site of CR infection for *K. pneumoniae* (48.7%) and *E. coli* (57.3%). Differences in the frequency of CR infection by infection site have also been reported in other studies [9, 23, 24].

The CR rates varied by the geographic regions. The Middle Atlantic region had the highest proportion of CR infections due to *K. pneumoniae* and *E. coli* (33.1%), while the New England region had a higher proportion of CR infections due to *P. aeruginosa* (85%). Other studies also observed the variations in frequency of CROs by hospitals and by region [22, 24, 25]. Although actual incidence cannot be estimated from this study, the regional differences observed in this study support the understanding that infection control of resistant pathogens needs to be based on local epidemiology.

As shown in this study, a majority of the CR pathogens were treated with multiple antibiotics, especially for *A. baumannii* and *P. aeruginosa* or for infections in the blood or respiratory infection sites. Even with combination treatment, patients with CR infections still had longer total hospital (total and after positive culture) and ICU stays, except among nonsurvivors with *A. baumannii* in blood and respiratory, where CR patients had shorter LOS after positive culture compared with CS patients and the median for CR in blood was 2 days after the culture was obtained. This further highlights the treatment challenges faced by clinicians today and the need for more effective antibiotics and more rapid information on antibiotic susceptibility.

A remarkably high number of different antibiotic combinations were used to treat these infections, especially for CR pathogens. This indicates that there is no generally accepted standard of care regimen for CR infections, which underlies the fact that there are few available antibiotics to treat CR infections, and those that may be available are associated with significant toxicity.

The authors believe that the key strength of this study is the large, geographically diverse data set that includes both detailed microbiologic data as well as patient-level information and outcomes. Unlike the CDC and other data sets that focus on hospital-acquired infections, the Premier Healthcare database includes both hospital-acquired and community-onset infections, many of which are healthcare related, thus better representing the full disease burden related to CR infections in US hospitals.

This study selected subjects based on microbiology results, not based on the discharge diagnosis, avoiding potential biases in the discharge diagnoses due to various reasons, including coding errors or reporting bias. The results of the microbiology and drug susceptibility testing, as well as the documented antibiotic treatments, provide a clear picture of the pattern of infections in acute care hospitals.

However, there are also several limitations in this study. First, case definitions were based on microbiological culture results that may underestimate or overestimate the number of infections. Although some culture results may represent colonization rather than true infection, this study only analyzed patients for whom the cultures had susceptibility testing performed and where their physician prescribed systemic antimicrobial therapy. In addition, focusing only on the 4 most common Gram-negative pathogens underestimates the total disease burden caused by CR infections.

Second, as microbiologic data were not collected from all hospitals in the Premier Network (teaching hospitals were more likely to provide microbiology data), there may be a reporting bias favoring a more complex patient population.

Due to the privacy concern by Premier, participating hospitals can only be identified by US census region. Therefore, it was not possible to estimate CR infection rates in smaller geographic areas, even though infections could vary from city to city and hospital to hospital. This study does identify regional differences in prevalence rates; however, it does not assess whether these findings are driven by individual institutions or episodic outbreaks within institutions.

This study only examined 2 Enterobacteriaceae, *E. coli* and *K. pneumoniae*, and 2 nonfermenters, *A. baumannii* and *P. aeruginosa*. Other nonfermenters, for example, *Stenotrophomas maltophilia,* which are highly carbapenem resistant [26, 27], may represent another significant source of disease caused by CR pathogens.

## CONCLUSION

This study showed that CR non-glucose fermenting Gram-negative bacteria, such as *A. baumannii* and *P. aeruginosa,* contribute to the greater disease and mortality burden when compared with CREs, such as *K. pneumoniae* and *E. coli*. This has been observed for the study as whole and for each census region. These nonfermenters contributed more than 80% of CR cases in our analysis of data over a period of 5 years. CR rates differed from region to region, but the predominance of CR nonfermenters over CREs was consistent. The geographic variability was greater among *K. pneumoniae* and *E. coli* than the nonfermenters. However, because the authors cannot determine specific population numbers by hospital, the incidence of CR infections cannot be estimated or compared. These data for the United States are in sharp contrast to most reports from Europe and Latin America, where CREs are predominant.

Because patients with CR infections had longer hospital and ICU stays, more antibiotic treatments, and a higher mortality rate, the CR infections likely represent a higher cost burden to the health care system. The development of new antibiotics should not only address CREs, but more importantly, it should address CR non-fermenters such as *P. aeruginosa* and *A. baumannii*.
